# Real‐Time High‐Definition Hyperspectral Endoscopy via Spatial‐Temporal Low‐Frequency‐Stochastic Spectral Encoding

**DOI:** 10.1002/advs.202517746

**Published:** 2026-01-15

**Authors:** Xiaowei Liu, Jiakang Shao, Julin Xiao, Chenying Yang, Jiahe Zhang, Xiaoyu Yang, Xiang Hao, Ying Gu, Xu Liu, Yizhou Tan, Ji Qi, Qing Yang

**Affiliations:** ^1^ Research Centre for Frontier Fundamental Studies Zhejiang Lab Hangzhou China; ^2^ Medical School of Chinese PLA Beijing China; ^3^ State Key Laboratory of Extreme Photonics and Instrumentation College of Optical Science and Engineering Zhejiang University Hangzhou China; ^4^ ZJU‐Hangzhou Global Scientific and Technological Innovation Center Hangzhou China; ^5^ Hangzhou Institute for Advanced Study University of Chinese Academy of Sciences Hangzhou China; ^6^ Department of Chemical Engineering and Biotechnology University of Cambridge Cambridge UK; ^7^ Department of Laser Medicine First Medical Center of Chinese PLA General Hospital Beijing China

**Keywords:** diffuse reflectance, hyperspectral endoscopy, low‐frequency stochastic filter, neural network, spectrum encoding

## Abstract

Hyperspectral endoscopy enables minimally invasive visualization of both structural and compositional information, offering promising potential for the accurate assessment of in vivo physiological and pathological conditions. However, current hyperspectral endoscopy suffers from low frame rate, hindering the clear capture of in vivo tissues in motion, restricting in vivo diagnostics, efficacy assessment, or risk monitoring during minimally invasive procedures. Here we propose a hyperspectral endoscopy by developing a spatial‐temporal spectral encoding approach based on low‐frequency stochastic filters combined with an encoding‐guided spectral attention network (ESANet) to reconstruct the hyperspectral image with low latency. A prototype system is developed to achieve real‐time frame rate (20 Hz), high‐definition resolution (full pixels), 67 spectral channels spanning 420–750 nm. It can overcome the continuous motion of in vivo tissue to provide hyperspectral images with fine superficial features, including capillary as small as around 37 µm in diameter, reveal the distinct spectra characteristics for diverse types of organs, and enable visualization of rapid and subtle compositional changes in two representative processes: photodynamic therapy and hepatic ischemia. With minimal hardware modifications, the proposed scheme provides a cost‐efficient and easily adaptable solution for hyperspectral endoscopy as well as broader application scenarios.

## Graphical abstract

A real‐time, high‐definition hyperspectral endoscopy is enabled by developing a spatial‐temporal spectral encoding approach based on low‐frequency stochastic filters combined with an encoding‐guided attention network. It provides hyperspectral image of in vivo tissue with fine superficial features, enables visualization of rapid and subtle compositional changes, and offers potential for accurate diagnosis and precise treatment guidance.

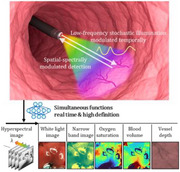

## 1 Introduction

Diffuse reflectance (DR) hyperspectral image (HSI) can provide an important insight into tissue structures and compositions with spatial details non‐destructively, which are useful to assess physiological and pathological conditions. It has been used to estimate the hemoglobin levels of the exposed tissues or skins, and to assess the tumor margin of the resected tissues in surgery [1, 2, 3, 4, 5, 6]. Equipping the endoscope with the hyperspectral imaging function to perceive structural and compositional changes of tissues can greatly benefit the endoscopy‐based screening, diagnosis and treatment. However, challenges remain in applying existing HSI acquisition approaches to endocavitary scenarios, which are characterized by limited space, continuous motion, and cross‐scale spatial features. The acquisition approaches based on spatially or spectrally scanning are slow in general. As a result, the obtained images often suffer from severe motion artifacts [7, 8, 9], resulting from natural respiration, gastrointestinal peristalsis, and procedural shaking. It is also difficult to image biological processes that are rapid changing such as vascular occlusion or vascular recanalization where blood vessel, flow and oxygen levels change within seconds. The approaches relying on division of focal plane (DoFP) image sensors are limited in terms of the number of spectral channels and spatial resolution. Compressive hyperspectral imaging approaches need careful modification with proper alignment to the optical path [10, 11], or a large encoding number (≥16 usually) via the DoFP or scanning schemes [12, 13, 14, 15, 16, 17], which also gives rise to issues in the fabrication cost, imaging speed, and spatial resolution for broad application in general clinical endoscopic settings. Consequently, hyperspectral imaging has so far been primarily limited to easily stabilized or resected tissues. A hyperspectral endoscopy offering spatial resolution and frame rate comparable to those of standard white light endoscopy (WLE), remains difficult to achieve, yet would offer substantial benefits for accurate diagnosis, risk monitoring, and treatment evaluation in the fields ranging from clinical medicine to biological research (Table S2).

Here, we propose a hyperspectral low‐frequency‐domain stochastic encoding endoscopy (HeldSee), suitable for real‐time high‐definition hyperspectral imaging of intracavity tissue in vivo, based on spatial‐temporal low‐frequency‐stochastic filtering combined with an encoding‐guided spectral attention network (ESANet) to reconstruct the HSI. HeldSee enables real‐time frame rate (20 Hz), high‐definition resolution (pixels number and spatial resolution equivalent to the standard WLE), 67 spectral channels covering the 420–750 nm range, and high spectrum accuracy (relative absolute error (RAE) < 5%) which has been validated on diverse types of organs. The high‐quality HSI provided by HeldSee can clearly reveal the fine texture features for in vivo tissues in continuous and deformable motion, including capillaries as small as around 37 µm in diameter. We also show that HeldSee enables spatially‐resolved detection of rapid and subtle compositional changes occurring within sub‐second intervals (that are challenging to detect using standard WLE and narrow‐band endoscopy (NBE)) in two representative processes: photodynamic therapy (PDT) and hepatic ischemia. HeldSee also possesses great potential for practical deployment on both rigid and flexible endoscopy, as it only involves alterations to the external endoscopic illumination module at the hardware level, which can be packaged as a compact module with a standard output port. These advantages make HeldSee a powerful, cost‐effective, and clinical translatable tool for minimally‐invasive diagnosis, treatment and biomedical studies with enhanced precision. Moreover, its spatial‐temporal low‐frequency‐stochastic spectral encoding design facilitates broader implementations in diverse active illumination imaging contexts, by incorporating modulated illuminators similar to the original configuration.

## 2 Principles of HeldSee

**FIGURE 1:**
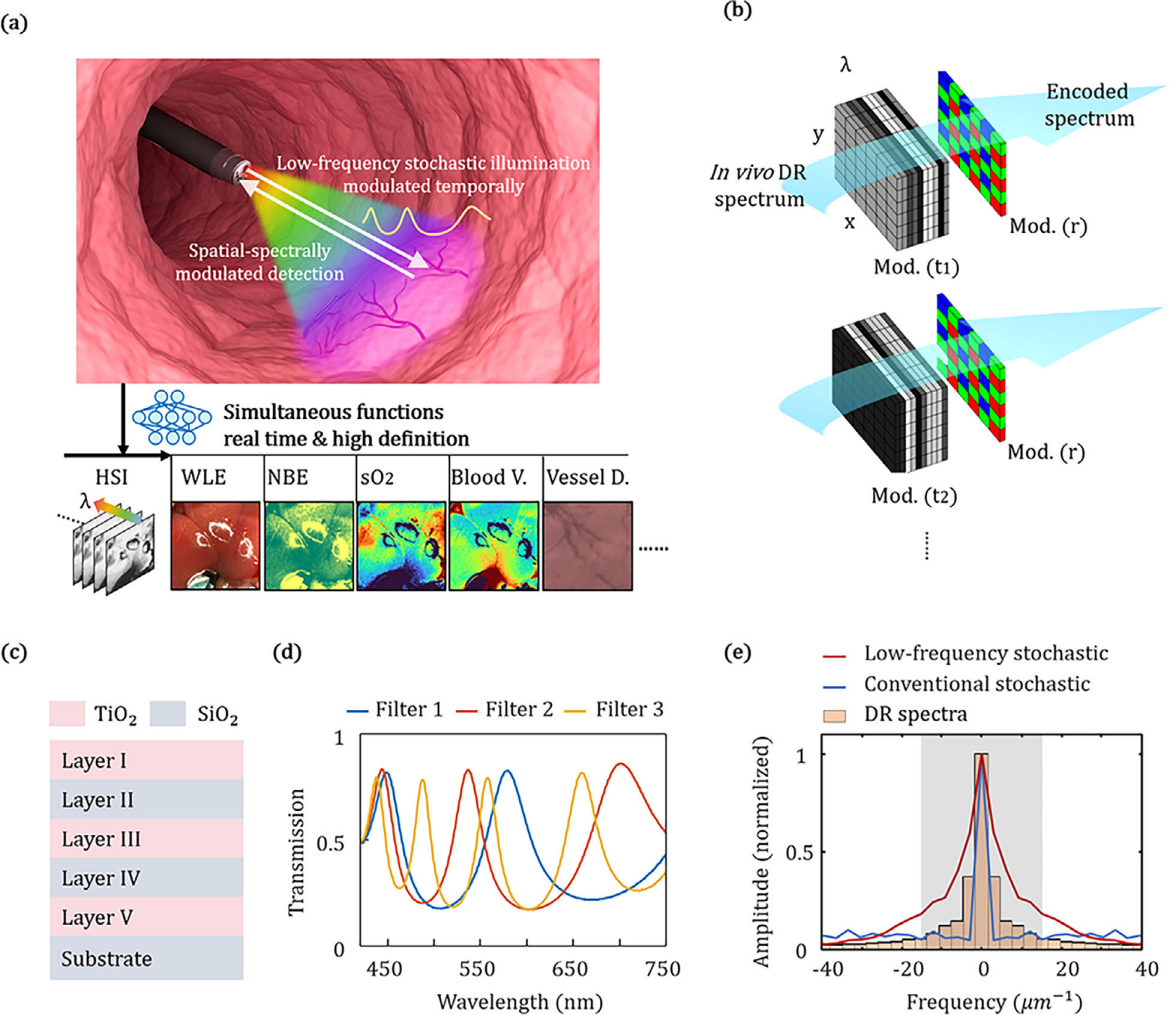
Schematic of the proposed HeldSee system. (a) A HeldSee distal end within a biological cavity, enabling the real‐time high‐definition HSI and multiple functional visualizations simultaneously. The DR spectrum of the in vivo intracavity are encoded by the temporal‐spectrally modulated illumination and the spatial‐spectrally modulated detection, and then decoded by a neural network. “Blood V.” denotes “Blood Volume”. “Vessel D.” denotes “Vessel Depth”. (b) Schematic of the encoding process in HeldSee. Mod. (t) denotes the temporally varied spectrum modulations with low‐frequency stochastic spectra; Mod. (r) denotes the spatially varied spectrum modulation. (c,d) The layer structure and the experimentally measured transmission spectra of the low‐frequency stochastic filters used in HeldSee. (e) Amplitude of the Fourier transform of the spatial‐temporal spectral encoding function based on the low‐frequency stochastic filters (red line), the conventional stochastic encoding function with random spectra (blue line), and the DR spectra (amplitude averaged between diverse tissue sample). A gray region shows the dominant range of the Fourier amplitude of the DR spectra (>5% of the zero‐frequency component).

The proposed HeldSee system consists of a light source with temporally modulated spectra and an endoscope equipped with a color imaging sensor. The light source sequentially generates illuminations with three low‐frequency stochastic spectra, as schematically demonstrated in Figure 1a, enabling temporal‐spectrally encoding. Meanwhile, RGB Bayer filter mask on the imaging sensor performs spatial‐spectrally encoding. To achieve high encoding efficiency with a small encoding number—crucial for simultaneously ensuring high spatial resolution and fast imaging speed—low‐frequency stochastic filters are used, based on the fact that tissue diffuse reflectance (DR) spectra are primarily dominated by low frequency components, with few sharp peaks. The transmission spectra of these filters are wide‐band, low in correlation, and their Fourier transforms are predominantly concentrated in the low‐frequency range. The synergy between rapid temporal modulation and the mainstream Bayer spatial mask offers key advantages for spectrum encoding, including real‐time performance, high definition and low cost. An ESANet algorithm is developed to reconstruct the HSI. The reconstructed HSI is informative, which can further support the multiple functional images including WLE and NBE, and visualizations of compositional and structural maps, simultaneously for accurate diagnosis and precise treatment guidance [18, 19, 20, 21, 22]. (Figure 1a). The total spatial‐temporal spectral encoding process is schematically illustrated in Figure 1b.

The intensity of the encoded image is the weighted sum of the Fourier components of the spectrum of the target sample, with the weight equal to the conjugate of the Fourier transform of the encoding function, as illustrated in (Equation 1) (The detailed deduction can be found in Part S11).

(1) $$I=\int E*\left(\nu \right)\cdot T\left(\nu \right)d\nu$$

in which E* is the conjugate of the Fourier transform of the encoding function; *T* is the Fourier transform of the spectrum of the target sample; *ν* is the frequency in the Fourier domain. According to (Equation 1), larger amplitude of the Fourier transform of the encoding function represents higher encoding weight on the target Fourier component at the corresponding frequency. Consequently, it is an efficient way to use the encoding function with large Fourier amplitude at the frequencies where the target sample dominates.

Wavelength‐scale thick filters are utilized to generate transmission spectra of low frequency and low correlation. The weakly‐confined Fabry‐Parrot cavity structure is utilized, which consists of five layers of SiO_2_ or TiO_2_ alternately stacked on a SiO_2_ substrate (Figure 1c). Designed thicknesses of the layers TiO_2_/SiO_2_/TiO_2_/SiO_2_/TiO_2_ are “60/94/240/94/60”, “60/94/320/94/60”, “60/94/560/94/60” (unit: nm), respectively. Figure 1d shows the experimentally measured transmission spectra of the three filters. The mean correlation coefficient between the three transmission spectra is 0.05 (Figure S5), which is a relatively low value to suppress the ill‐condition in the reconstruction problem. The final spatial‐temporal spectral encoding function is the product of the transmission spectra of the filters in illumination and that of the Bayer mask in detection (Equation 2).

(2) $$e_{i,j}\left(x,y\right)=f_{i}\left(\lambda \right)\cdot d_{j}\left(\lambda \right)\cdot s\left(\lambda \right)$$

where *x* and *y* denote the spatial coordinates; *λ* denotes wavelength; *f* denotes the transmission spectra of the filters; *d* denotes the transmission spectra of the Bayer mask; *i* denotes the index of spectrally modulated illumination and *j* denotes the color channel in detection (*i* = 1, 2, 3 and *j* = 1, 2, 3); *s* denotes the spectrum of the light source without filter.

To demonstrate the high encoding efficiency, we have made Fourier transform for the DR spectra of diverse tissue sample, as well as for the spatial‐temporal spectral encoding function based on the low‐frequency stochastic spectra and the conventional stochastic encoding function with random spectra (Figure S15) for comparison [15, 16, 17]. The Fourier components of the DR spectra primarily concentrated within the range of 18 µm^−1^ (referred to as the dominant range, highlighted by the gray region in Figure 1e), with the Fourier amplitude outside this range dropping below 5% of the zero‐frequency component. The Fourier amplitude of the conventional stochastic spectra is uniformly distributed across both this dominant range and high frequency range, whereas the spatial‐temporal spectral encoding function used in HeldSee exhibits a significantly larger amplitude in the dominant range, leading to more efficient encoding (Figure 1e).

In implementation, the temporal‐spectrally modulated light source can be designed with a standard output port compatible with flexible or rigid endoscope structure, making this approach an adaptable solution (Figure S1). The outputs of three white light LEDs first pass different stochastic filters of low‐frequency and low‐correlation, and then are separately focused into the input ports of a 3‐in‐1 fiber bundle for beam combination. As only three modulations are utilized in the HeldSee light source for one cycle which can be switched with low delay, the in vivo tissue motion can be effectively overwhelmed. The imaging sensor is synchronously triggered to capture one image during the illumination period of each LED, resulting in three raw images per cycle for reconstruction.

**FIGURE 2:**
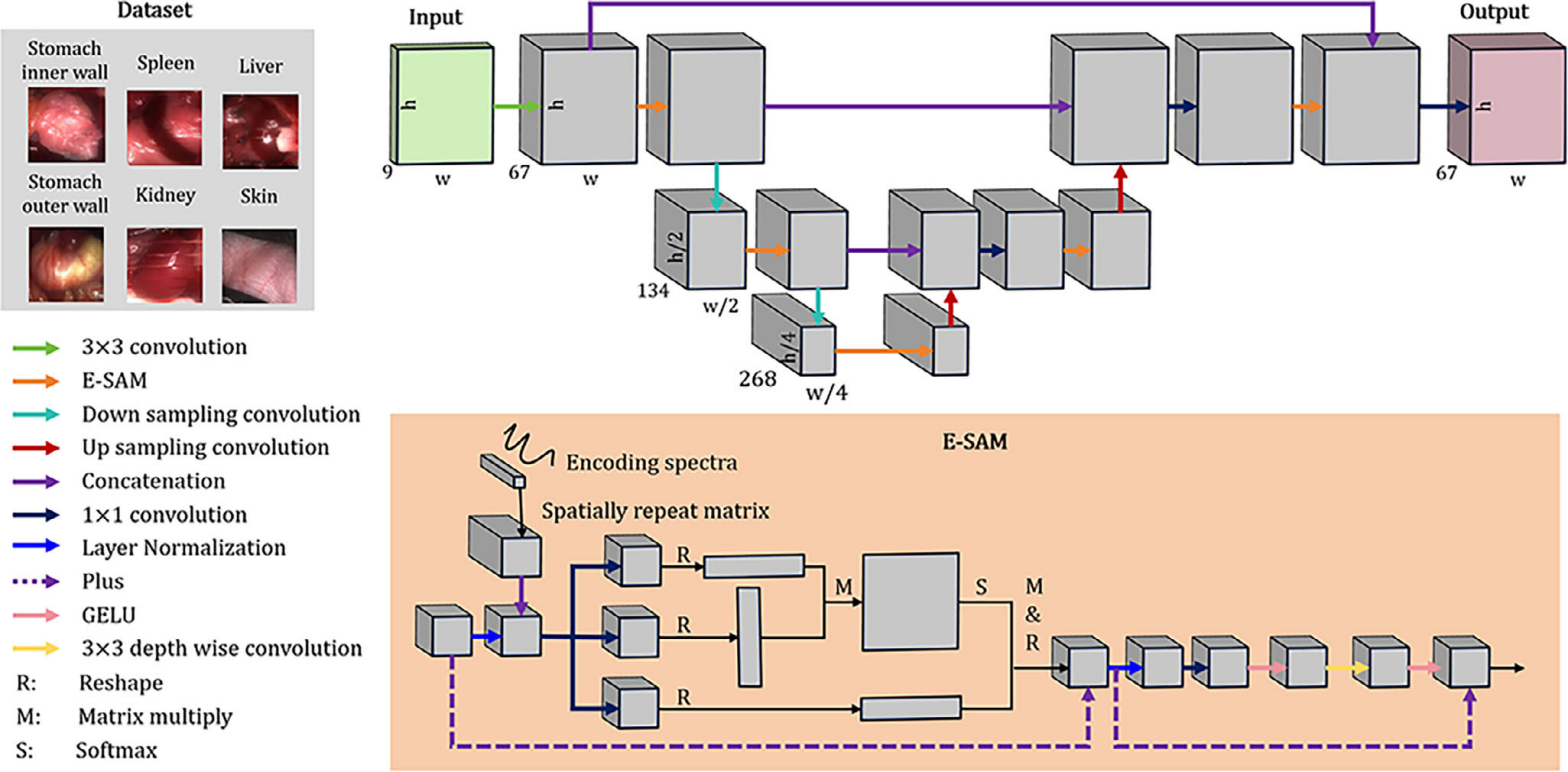
Reconstruction algorithm. Framework of the proposed ESANet with an E‐SAM module. Top‐left inset: composition of the dataset used to train the network.

An ESANet is developed to reconstruct the HSI from the real‐time, pixel‐level encoded images with low‐latency, as illustrated in Figure 2. The network employs a U‐Net‐style encoder decoder architecture, with an encoding guided spectral attention module (E‐SAM), to reconstruct HSI from the measurement. Compared to the conventional attention modules, the integration of the encoding spectra for guidance can increase the spectral reconstruction accuracy (Figure S10). The HSIs used to train the network are practically collected from living mice, including several types of organ: liver, spleen, kidney, stomach inner wall, stomach outer wall and ear skin (top‐left inset in Figure 2). With the spectra of the illumination modulation and the spectral responses of the detection of the HeldSee system calibrated (Calibration processes and results can be found in Part S2), the mapping pairs to train the network can be generated according to (Equation 3), with the detection noise and the intensity variation between different modulated illuminations considered. Details of the dataset and network training parameters are in Part S4.

(3) $$I_{i,j}\left(x,y\right)=V_{i}\cdot \int e_{i,j}\left(x,y\right)\cdot t\left(x,y,\lambda \right)d\lambda +N$$

where *I_i,j_* denotes the intensity of the encoded image at spatial location of (x, y); *e_i,j_* denotes the encoding spectra, defined in Equation 2; *t* denotes the DR spectrum of tissue; *V_i_* denotes the random intensity variation of the modulated illuminations; *N* denotes the noise.

## 3 Performance of HeldSee

**FIGURE 3:**
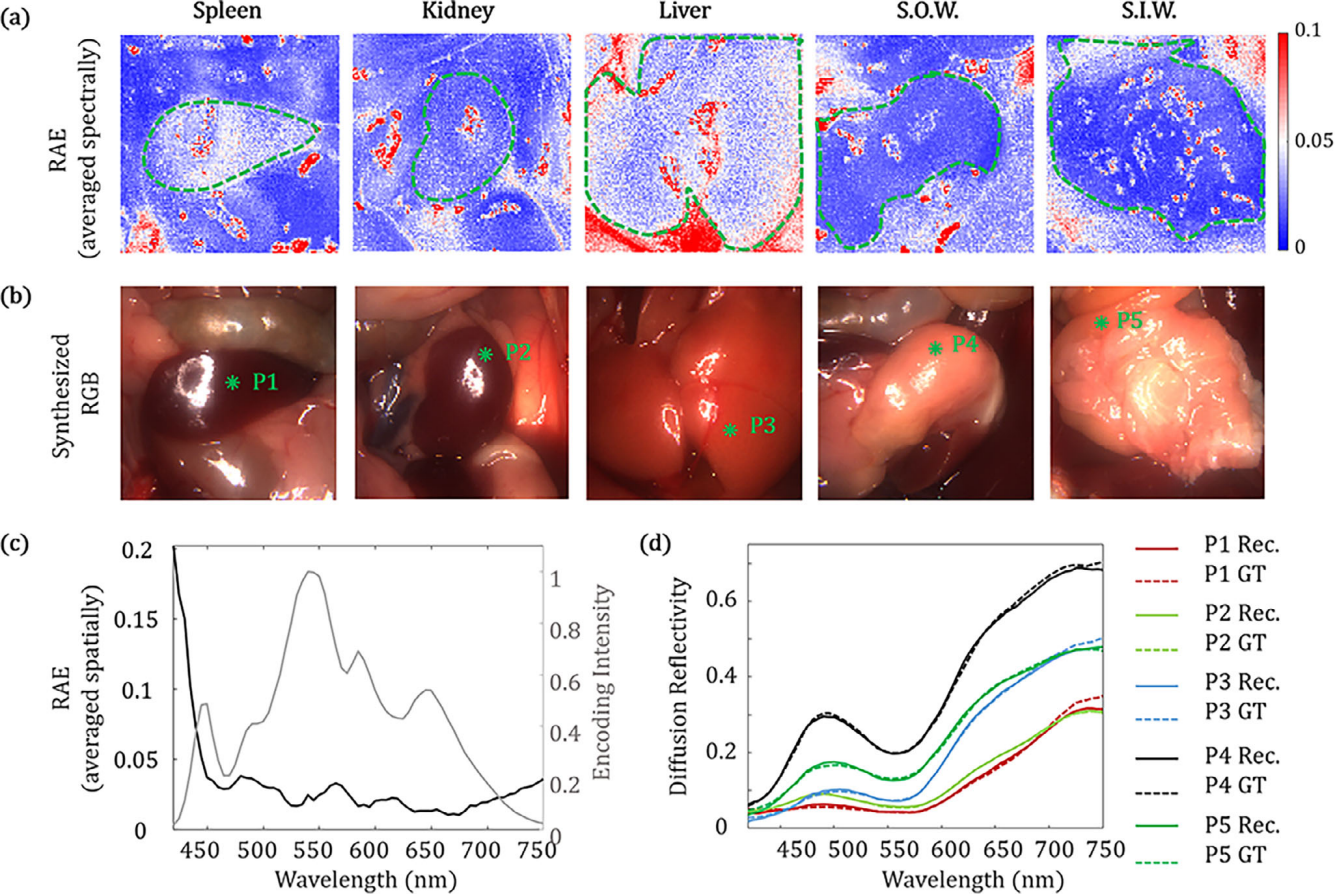
The spectrum accuracy achieved by HeldSee for diverse types of organs. (a) The spatial distribution of the RAE demonstrated on the simulated measurements, which contains 5% detection noise and 5% intensity variation of between different modulated illuminations, for diverse types of organs. The RAE is averaged spectrally for each pixel. (b) The RGB view sight synthesized from the GT HSI cube. (c) Left axis: The RAE at different wavelengths for diverse types of organs, which is spatially averaged in the regions circled by the green dash lines in (a). Right axis: Encoding intensity achieved by the sum of all the encoding spectra. (d) The reconstructed spectral results of the HeldSee (solid lines) and the GT (dash lines) for points labeled with green asterisks in (b). S.O.W. denotes “stomach outer wall”, S.I.W. denotes “stomach inner wall”.

HeldSee can reveal the distinct spectral characteristics for various abdominal organs, which is helpful to the endoscopy‐based identification and navigation. The spectrum reconstruction accuracy of HeldSee was validated on diverse types of organs, including stomach outer wall, stomach inner wall, liver, kidney and spleen. Figure 3a shows the RAE averaged in the spectral dimension, between the ground truth (GT) and the HeldSee result reconstructed from the simulated measurement with noise level of 5% and illumination intensity variation of 5%. Figure 3b shows the corresponding RGB images synthesized from the GT, presenting intuitive view sights. The spectrally averaged RAE is confined within 5% across the test organs (organ boundaries are plotted using green dash lines), except some regions overexposed with strong specular reflection (Figure 3a). We also analyzed the reconstruction error at different spectral band as shown in Figure 3c, where the reconstruction RAE is averaged among all the test organs in Figure 3a. We find the RAE is confined within 5% in the range from 450 nm to 700 nm, while increases heavily at the short wavelengths. This could be owing to the encoding intensity (Equation 2) is low at the two ends, which is plotted at the right axis in Figure 3c. Moreover, as the absorption is more severe in the shorter wavelength, the DR intensity is low and thus more vulnerable to noise. Figure 3d plots spectra achieved by HeldSee for the five types of organ tissues at the green asterisks in Figure 3b, in comparison to the GT. The HeldSee reveals the varied spectra across different organs, which coincide well with the GT for most wavelengths. We also compared the HeldSee results reconstructed from the experimental measurements with the results acquired by a wavelength‐scanning approach. The results also match well which can be found in Part S5. The spectral resolution of HeldSee system has been evaluated using two criteria: (1) the closest double peaks it can resolve, and (2) the bandwidth of the measured profile of a narrow peak. It can resolve double peaks with a 20 nm peak‐to‐peak separation and achieve bandwidths slightly below 20 nm for narrow single peaks, demonstrating that the spectral resolution of HeldSee system exceeds 20 nm. The testing methods and the achieved results are presented in detail in Part S3.

**FIGURE 4:**
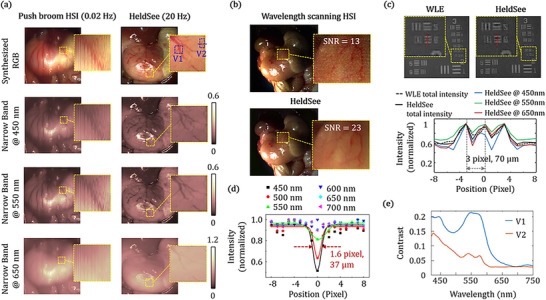
Imaging performance of HeldSee on frame rate, spatial resolution and SNR. (a) Comparison of the HSI results achieved by HeldSee and the spatial scanning approach (push broom) for mouse outer stomach walls in continuous motion caused by peristalsis and breath. The synthesized RGB images and exemplar spectral images at 450, 550, and 650 nm are shown. Regions containing capillaries is enlarged for a clearer show. (b) Comparison of the HSI results shown in RGB format achieved by HeldSee and the wavelength‐scanning approach for a motionless mouse outer stomach walls at dim condition. Calculated SNRs are labelled on top of the magnified view sight. (c) Spatial resolution evaluation for HeldSee in comparison with WLE, using the USAF 1951 resolution test chart placed at 25 mm working distance. Plotted along the red dashed line for the sixth element of the third group are the total intensities of WLE and HeldSee, and the HeldSee intensities at three spectral bands. (d) Spatial resolution evaluation at in vivo condition for HeldSee. The intensity distributions across a thin capillary (∼ 37 µm thick) shown along the blue dash line in (a) are plotted at 450 nm to 700 nm, with a step of 50 nm. (e) Image contrast of regions containing deeper vessel (V1 in (a)) and superficial capillary (V2 in (a)) at different wavelength.

Then we show the advantage of the proposed HeldSee on the frame rate to photograph the intracavity in vivo organs in continuous and deformable motion. The HeldSee is implemented in the form of a rigid endoscope to simulate laparoscopy. The experimental setup of the prototype is shown in Figure S2. Figure 4a shows the hyperspectral endoscopy images of the stomach outer walls (for mice). In the images shown in the left column in Figure 4a, which are achieved by replacing the camera of a commercial endoscope to a conventional push broom hyperspectral camera (GaiaField‐V10, frame rate: 0.02 Hz), severe motion blur occurs and it is difficult to observe the fine capillaries. While with the HeldSee system, as the 3 LEDs and the synchronous controlled camera state can be switched within microseconds without mutual interference, it is possible to record the raw image group with a speed of several tens Hertz, which could overwhelm most biological motion in the tissue level (A test of 66 Hz is in Part S2). The developed algorithm can reconstruct hyperspectral image of low error from the motion‐artefact‐free encoded images (Part S4). Here a 20 Hz frame rate is adopted in the in vivo experiments, which could ensure no motion artefacts and high signal‐to‐noise‐ratio (SNR) of each encoded image. The reconstructed HSI shows clear capillaries structure without blur (right column in Figure 4a). A video showing HeldSee image of the stomach in motion is included in the Supplementary materials (Video S1). The reconstruction time to generate a HD HSI based on an AMD Ryzen 9 5900 × 12‐Core CPU and NVIDIA GeForce RTX 3090 GPU platform is 0.2 s.

To validate the high sensitivity of HeldSee, we compared the SNR at the dim condition between the HeldSee and the conventional wavelength‐scanning method. For the sake of fairness, the same camera is used in the two approaches while only the illumination modulation way is different. In the conventional method, narrow band illumination modulation is utilized, with its central wavelength scanning from 420 to 750 nm, and the peak intensities are mostly larger than the intensities used in the HeldSee at the corresponding central wavelengths (The realization of the two approaches as well as the intensity comparison can be found in Part S5). For in situ comparison, a motion‐less stomach outer wall of a mouse that just died is used as the sample, to avoid the motion blur in the wavelength‐scanning approach. The HSI based on the HeldSee and the wavelength‐scanning approach are shown in Figure 4b in synthesized RGB format. The comparison on 2D images at each spectral channel can be found in the Part S5. The SNR is calculated as the ratio of the mean intensity to the standard deviation within a region containing few blood vessels. HeldSee enables a SNR almost twice that achieved using the wavelength‐scanning method, as labelled in Figure 4b.

The spatial resolution of the HeldSee is evaluated in comparison to the classical WLE, with the same color camera (MER2‐230‐168U3C, 1920 × 1200 Full HD, Daheng) and endoscope body, using an USAF 1951 spatial resolution test chart as the object. Figure 4c shows the imaging intensity achieved using WLE and HeldSee, respectively, with the resolution test chart placed at the 25 mm working distance. The minimum units that can be distinguished by WLE and HeldSee both are the sixth element of the third group (period: 70 µm), whose peak‐to‐peak distance occupies 3 pixels in the imaging plane, demonstrating the spatial resolution of HeldSee is comparable to the classical WLE to enable the high‐definition imaging. The spatial resolution of HeldSee has also been evaluated for the in vivo case, using the imaging linewidth of an ultra‐thin capillary, as shown in Figure 4d. The imaging linewidth and contrast both change with the wavelength. The linewidth is ∼1.6 pixel at 450 nm (representing an object size around 37 µm), demonstrating the high‐definition imaging quality enabled by HeldSee to reveal the fine texture features of in vivo tissues in motion.

The wavelength‐dependent contrast provided by HeldSee can reveal depth‐related characteristics of blood vessels, enabling the extraction of more structural information. Due to the stronger absorption and scattering of short wavelengths within tissue, the peak of contrast shifts toward longer wavelengths as the vessel depth increases. Figure 4e plots the image contrasts at different wavelengths, for two small regions V1 and V2, labeled by two blue squares in Figure 4a. For V1 contains thicker blood vessels generally located deeper within the mucosal layer, the highest image contrast appears around 545 nm. For V2 mainly contains superficial capillary, the image contrast is apparently higher at the shorter wavelength of 425 nm. This contrast‐wavelength relationship coincides with the functional NBE that usually selects near‐UV blue illumination to enhance the contrast of superficial capillary and green illumination for the deeper vessels.

## 4 Applications of HeldSee on Monitoring of the Rapid Physiological Process

**FIGURE 5:**
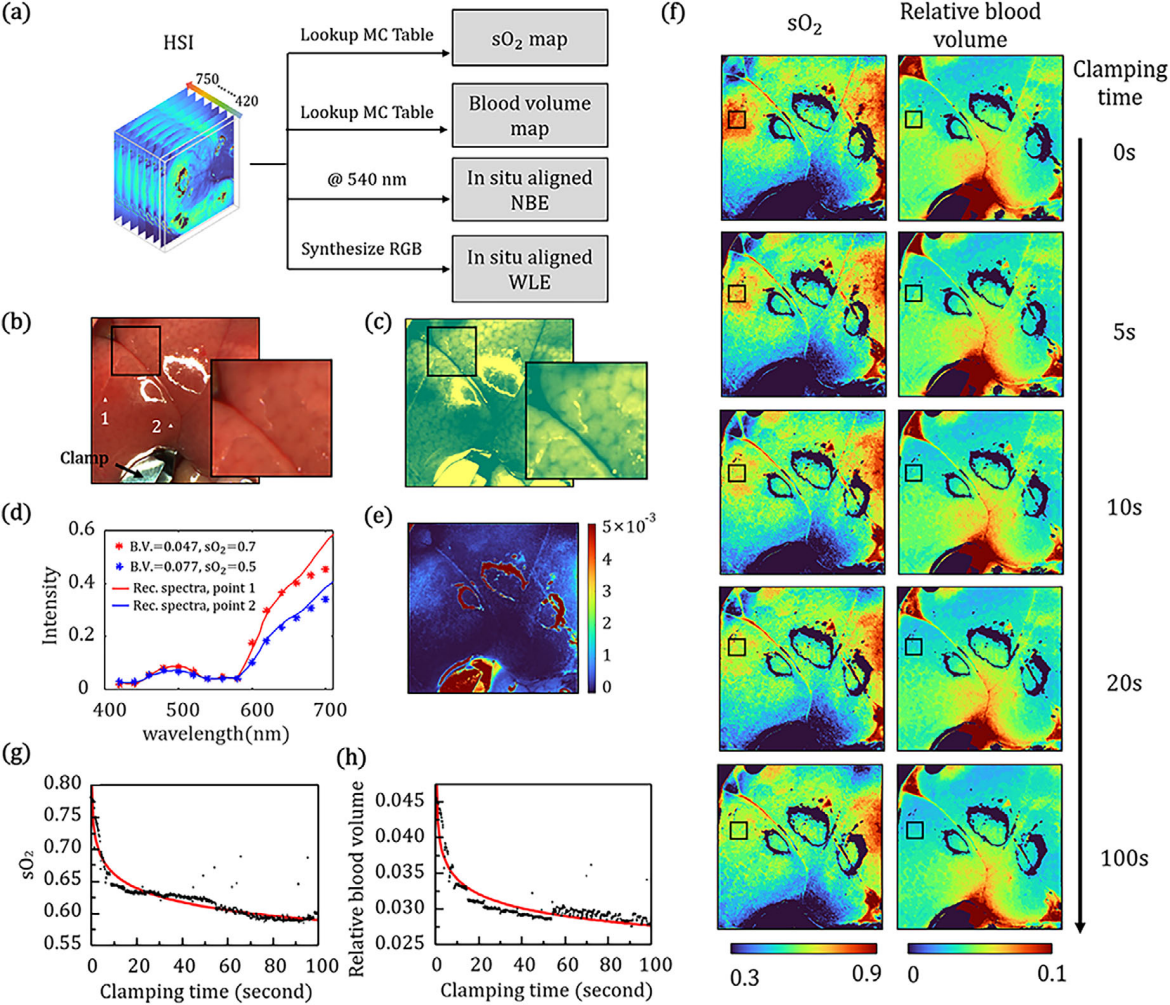
Application of HeldSee on liver ischemia monitoring. (a) Available functions of HeldSee based on its high‐definition and real‐time HSI acquisition performance. “MC” denotes “Monte Carlo”. (b,c) WLE image (b) and NBE image (c) of a clamped liver in ischemia, generated from the HeldSee image. The regions in the black squares are magnified for a clearer view. (d) The comparison between the spectra achieved by HeldSee (solid line) and the DR spectra calculated based on the MC model (asterisk) with the sO_2_ and relative blood volume determined using a lookup table method, at points labeled by the white dot in (b). “B.V.” denotes “relative blood volume”. (e) The spatial distribution of the deviation (MSE) between the spectra achieved by HeldSee and the spectra calculated based on the MC model with the sO_2_ and relative blood volume determined for each pixel using a lookup table method. (f) The spatial distribution of sO_2_ and relative blood volume at different time from the start of the clamping. (g) The change of sO_2_ and relative blood volume with the clamping time, in the region labelled by the black square in (f).

With the hyperspectral information provided by HeldSee, the content of the biological composition can be analyzed in vivo with high spatial resolution, free of motion blur, and with a rapid response, which are good indicators of the physiological state, and can be used to enhance the diagnosis and screening capability of endoscopy in clinics, as well as to study the fast physiological changes in biomedicine researches [23]. The compositional graphs showing the oxygen saturation and the relative blood volume can be derived from the HSI with a lookup table, established based on the Monte Carlo (MC) light transport simulation method [24]. On the other hand, with HeldSee, the image at the specific spectral channel can be extracted to simulate the WLE and NBE image, no need to add additional light sources. The generated WLE or NBE images are in situ aligned with the compositional graphs, which is helpful to make cooperative diagnosis. In current HeldSee, the spectral channel of 415 nm is uncovered as the intensity of the white LED used in the configuration is relatively low below 420 nm, and the spectral channel of 540 nm is extracted for the NBE show. The available functions of HeldSee are demonstrated in Figure 5a.

First, we have used the HeldSee to monitor the occurrence of ischemia, a phenomenon caused by the imbalance between tissue oxygen supply and demand, which is a core inducer of myocardial infarction, acute liver injury or organ transplantation failure, etc. The pathophysiological characteristics of ischemia in tissue or organ has significant spatial‐temporal specificity, however, traditional monitoring methods (like ultrasound, CT, or angiography) suffer from limited spatiotemporal resolution or risk of invasion, etc. HeldSee, which can derive the blood characteristics from the DR with real‐time frame rate and high spatial resolution, is a possible non‐destructive approach to promptly detect the onset of ischemia in the clinical surgery and the biomedicine research.

In the experiment, the hepatic pedicle of a mice liver is clamped using vascular clamp, and the caused ischemia response is monitored using HeldSee with a frame rate of 20 Hz. Figure 5b shows the synthesized RGB image, analogizing to the classical WLE view. Figure 5c shows the image at the channel of 540 nm, analogizing to the classical NBE view. Contrast of the tissue texture can be enhanced in the generated NBE view (Comparison between WLE and NBE with the same colormap can be found in Part S8). A lookup table is established between the DR spectrum and the content of oxyhemoglobin and deoxyhemoglobin, based on the MC method (Details on the tissue model can be found in Part S9). Then the distributions of blood oxygen saturation (sO_2_) and the relative blood volume in the ischemia process can be achieved by identifying the nearest spectrum in the established table based on a least square method. Two exemplar points in Figure 5b are chosen to show the HeldSee spectra and the DR spectra calculated based on the MC model with the matched sO_2_ and relative blood volume (Figure 5d). We use mean square error (MSE) to estimate the deviation between the two spectrums, as shown in Figure 5e, in which the MSE is below 0.001 for most pixels except for those overexposed areas with high specular reflectance.

HeldSee enables spatially‐resolved detection of rapid compositional changes. Changing of sO_2_ and relative blood volume distributed in the complete FOV are shown in Figure 5f, at five time points (0s, 5s,10s, 20s and 100s) since the hepatic pedicle was clamped. As demonstrated in Figure 5f, the sO_2_ and the relative blood volume decrease with the clamping time, and the change speeds vary with spatial location, owing to the interruption of blood and oxygen supply, vascular collapse and circulatory disorders caused by mechanical obstruction. The mean values of sO_2_ and relative blood volume in a small region (black square in Figure 5f) are plotted in Figure 5g,h, quantitatively illustrating localized changes over time. A dramatic drop occurs within the first 10 s due to rapid clamping of the hepatic pedicle. Changes can be observed within sub‐second, thanks to HeldSee's real‐time feedback capability. While the onset and progression of ischemia with spatial details are difficult to capture using conventional hyperspectral endoscopy relying on a scanning procedure. A video showing the continuous dynamic changes of the sO_2_ and the relative blood volume in ischemia process is included in the Supplementary materials (Video S2).

Then we have used the proposed HeldSee approach to monitor the progression of PDT. PDT is a light‐based therapeutic modality that employs photosensitizers activated by specific wavelengths to induce cytotoxic effects, which can cause oxygen depletion and vascular occlusion. Due to the tumor‐selective accumulation of photosensitizers and the precise spatial control enabled by light exposure, PDT offers potential advantages in oncological applications, such as reduced collateral damage and enhanced therapeutic selectivity [25]. However, the degree of photodynamic effect exhibits significant individual variability and tissue heterogeneity, and at present, the treatment dosage mostly relies on doctors’ clinical experience, which limits its application promotion. Real‐time, quantitative monitoring of the photodynamic progression intraoperatively is essential to optimizing light dose, avoiding normal tissue damage and predicting treatment response and thereby achieving the goal of precise treatment. With real‐time feedback and quantitative analysis of blood and oxygen parameters, HeldSee possesses significant potential for guiding precision treatment in PDT.

**FIGURE 6:**
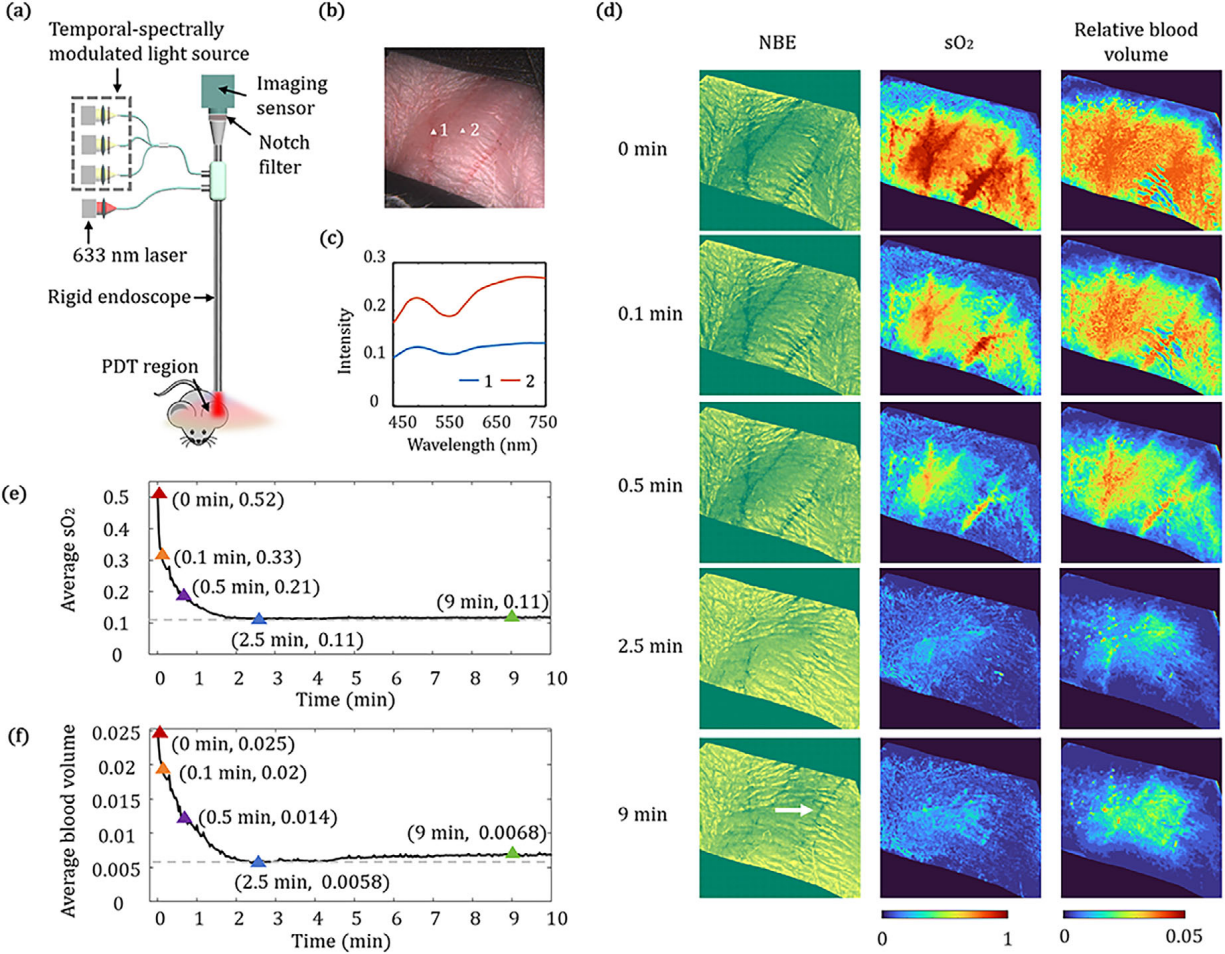
Application of HeldSee on monitoring the photodynamic process. (a) Schematic diagram of the HeldSee with photodynamic function, which includes laser and notch filter in the configuration. (b) Color‐correct WLE view of a mouse ear under photodynamic effect, generated from the HeldSee image. (c) Reconstructed complete spectra at white points in (b). (d) Generated NBE view, and the sO_2_ and the relative blood volume distribution at 5 time points from the start of the photodynamic effect occurring. A white arrow indicates the restored vessel. (e‐f) The changes of sO_2_ and relative blood volume of the mouse ear since the photodynamic effect occurs. The values at the 5 time points same as (d) are marked out.

For the endoscope with the function of PDT, a laser is equipped to stimulate the photodynamic effect and a notch filter is added in the imaging path to remove the laser from the captured images, as schematically shown in Figure 6a. Here we use the 633 nm laser for demonstration, which is a classical wavelength used in PDT. In experiment, the laser power density is 130 mW/cm^2^, which is a classical choice in clinics. A balb/c mouse aged 6–8 weeks was used, and HPD at a dose of 20 mg/kg was injected through the tail vein, and PDT was performed 30 min later on its ear with visible blood vessels.

Color bias that arises in conventional WLE images during PDT can be effectively avoided in HeldSee‐synthesized WLE images. The DR spectrum information suppressed by the notch filter can be restored by HeldSee to generate a color‐accurate image (Figure 6b). Figure 6c shows the reconstructed complete spectra of two exemplar points in the FOV labelled by the white triangle in Figure 6b.

HeldSee can detect the rapid and subtle compositional changes that are difficult to discern with the standard NBE and WLE. As demonstrated in Figure 6d are the distributions of sO_2_ and relative blood volume, as well as the NBE views reflecting the vascular morphology, at various time points after the laser was turned on. During the initial 0.1 min, dramatic changes are observed in the sO_2_ and relative blood volume graphs generated by HeldSee, whereas only slight alterations exhibit in the NBE view. A video showing the continuous dynamic changes of the sO_2_, the relative blood volume and vascular morphology during the PDT is included in the Supplementary materials (Video S3).

Figure 6e,f presents the change of the sO_2_ and the relative blood volume with time, which are spatially averaged in the FOV, for a quantitative analysis. The apparent decrease of the sO_2_ and relative blood volume continues about 2.5 min, and the variation becomes relatively stable afterward. Interestingly, the sO_2_ barely recovers while the relative blood volume gradually recovers a little (as shown by the images with a laser applying time of 9 min in Figure 6d) and some of the disappeared vessels were restored (as indicated by the white arrow in Figure 6d). The oxygen is continuously consumed by the photodynamic effect and thus cannot replenish, while the flowing blood repeatedly washes the blood vessels, which may induce the detachment of produced micro‐thrombi, leading to the vessel recanalization [26, 27]. These findings highlight HeldSee's ability to provide timely visual feedback during the course of PDT.

## 5 Discussion

The data compression that happens in the spectral encoding process, along with the wide‐band modulation with high speed and large luminous flux, allow for a hyperspectral endoscopy significantly outperforms the traditional approaches based on the narrow‐band‐splitting detection, in terms of the frame rate, spatial resolution and SNR [28, 29, 30, 31, 32, 33, 34]. Compared with the previously reported compressive hyperspectral imaging methods usually require dozens of modulations, only three temporal modulations are required in HeldSee, enabling real‐time, pixel‐level spectrum encoding and cost‐effective, easily‐deployable realization. Low‐frequency stochastic filters enable efficient encoding in the dominant frequency range of the DR spectra with small encoding number. Comparison on the spectrum reconstruction accuracy between low‐frequency stochastic and conventional stochastic modulation can be found in Part S6.

The high spatial‐temporal resolution and the high spectrum accuracy of HeldSee make it a good tool for the endoscopy‐based navigation, diagnosis and screening etc. HeldSee can reveal the distinct spectra characteristics for diverse types of organs in vivo with validated high spectrum accuracy. The high‐quality HSI provided by HeldSee can clearly reveal the fine texture features of in vivo tissues in motion, and can be leveraged to detect the rapid and subtle compositional changes on the sub‐second scale that are difficult to discern with the standard WLE or NBE. HeldSee requires only alterations to the external light source module of the endoscope and thus is compatible with both rigid and flexible endoscopes, facilitating regulatory approval and clinical translation.

The spectral range of HeldSee is currently limited by the white light source used in our prototype, which covers 420 nm to 750 nm. With hyperspectral information in such a range, it can enable efficient quantitative detection for hemoglobin, and provides graphs of oxygen saturation and the relative blood volume. Analyzing additional components, such as water and fat, requires a light source with broader spectral range that extends into the infrared region. Part S10 presents the dependence of the composition calculation accuracy on the spectral range for different components. On the other hand, more modulation number would be required to maintain the spectral resolution and spectral reconstruction accuracy across a broader spectral range.

For the specific higher‐level tasks, the best modulation function can be further explored, using an end‐to‐end optimization approach. This requires the doctor to participate more to propose the specific application and provide the labelled data. Because the modulation function can be flexibly tailored by replacing the filters with a plug‐and‐play manner. In the future, a versatile set of filter combinations for illumination modulation can be engineered as an accessory to support various advanced tasks, potentially making the HeldSee more cost‐effective, compact, flexible, and functional for clinical use. Furthermore, the spectral encoding scheme is adaptable to various active illumination imaging scenarios, such as industrial diagnostics, safety monitoring etc., by incorporating modulated illuminators similar to the original configuration.

To increase the reconstruction accuracy, one choice is to use more encoding. However, increased modulation introduces challenges, such as difficulty in achieving consistent illumination intensity distribution and reduced SNR of the individual encoded image due to shorter exposure times required to maintain frame rate. These factors, in turn, could reduce the reconstruction accuracy. The reconstruction accuracy with more modulation at different illumination consistency and noise ratio is presented in Part S7. Consequently, it is important to comprehensively evaluate practical factors such as the available light source energy, beam combining quality, expected frame rate, and cost when considering further upgrades.

One limitation of the HeldSee method is its scene dependence. As the dataset used to train the network only includes the spectrum of mouse organs, its spectrum accuracy could decrease for other types of biological tissues. While the procedure of this approach, including the collection of the effective dataset for in vivo sample in motion, the network training and prediction, can be transferred to the clinical scenes. By the way, as endoscopes are generally purpose‐specific, the demands for generalization could be lowered to some extent.

## 6 Conclusion

In this work, we have presented a new hyperspectral endoscopy, HeldSee, based on the spatial‐temporal spectral modulation with low‐frequency stochastic filters for spectrum encoding and AI‐assisted decoding. It enables real‐time frame rate, high‐definition spatial resolution (comparable to classical WLE) and high spectrum accuracy, which shows significant potential to benefit the endoscopy‐based navigation, diagnosis, and screening etc. It can overcome the continuous motion of in vivo tissue to provide HSI with fine superficial features, reveal the distinct spectra characteristics for diverse types of organs, and provide prompt feedback with spatial details for intraoperative monitoring. It possesses advantages of low cost and good compatibility with current clinical endoscope structure owing to the spectrally modulated illumination source can be packaged as a compact module with standard output port. In summary, the proposed HeldSee architecture is expected to bring widespread benefits to clinical practice, paving the way for non‐invasive, intelligent, and accessible healthcare solutions.

## Conflicts of Interest

The authors declare no conflicts of interest.

## Ethics Declarations

All in vivo experiments were approved by the Laboratory Animal welfare and Ethics Committee of Zhejiang University (No. 30838).

## Supporting information




**Supporting File 1**: advs73761‐sup‐0001‐SuppMat.pdf.

**Supporting File 2**: advs73761‐sup‐0002‐VideoS1.avi.

**Supporting File 3**: advs73761‐sup‐0003‐VideoS2.avi.

**Supporting File 4**: advs73761‐sup‐0004‐VideoS3.avi.

## Data Availability

The data that support the findings of this study are available from the corresponding author upon reasonable request.
