# Supplementary material for: Real‐Time High‐Definition Hyperspectral Endoscopy via Spatial‐Temporal Low‐Frequency‐Stochastic Spectral Encoding
Source: Adv Sci (Weinh). 2026 Jan 15;13(16):e17746. doi: 10.1002/advs.202517746 (PMC13042850; doi:10.1002/advs.202517746)
Supplement: Supplementary file 1 — Supporting File 1: advs73761‐sup‐0001‐SuppMat.pdf. [file ADVS-13-e17746-s002.pdf]

Supplementary information:

Real-time high-definition hyperspectral endoscopy via spatial-temporal low-frequency-stochastic spectral encoding

Xiaowei Liu,[1, †] *Jiakang Shao,*[2, †] *Julin Xiao,*[1, 3, 4] *Chenying Yang,*[5] *Jiahe Zhang,* [4] *Xiaoyu Yang,*[3, 4, 6] *Xiang Hao,*[3, 4] *Ying Gu,*[7] *Xu Liu,*[3, 4] *Yizhou Tan,*[7, *] *Ji Qi,*[1,*] *and Qing Yang*[3, 4, *]

[1] Research Centre for Frontier Fundamental Studies, Zhejiang Lab, Hangzhou 311121, China

[2] Medical School of Chinese PLA, Beijing 100853, China

[3] State Key Laboratory of Extreme Photonics and Instrumentation, College of Optical Science and Engineering, Zhejiang University, Hangzhou 310027, China

[4] ZJU-Hangzhou Global Scientific and Technological Innovation Center, Hangzhou 311215, China

[5] Hangzhou Institute for Advanced Study, University of Chinese Academy of Sciences, Hangzhou 310024, China

[6] Department of Chemical Engineering and Biotechnology, University of Cambridge, Cambridge CB3 0AS, UK

[7] Department of Laser Medicine, the First Medical Center of Chinese PLA General Hospital, Beijing 100853, China

[†] The authors contribute equally to the work.

Contents

Part 1 Realization of the HeldSee system

Part 2 Calibration of the prototype

Part 3 Spectral resolution of the system

Part 4 Details of collected dataset and the reconstruction network

Part 5 Signal-to-noise ratio comparison between HeldSee and conventional scanning approach

Part 6 Reconstruction accuracy with low-frequency and conventional stochastic filters

Part 7 Reconstruction accuracy with increased modulation number

Part 8 Narrow band imaging function of HeldSee

Part 9 Monte Carlo calculation model

Part 10 Dependence of the composition calculation accuracy on the spectral channel number

Part 11 Encoding analysis in the Fourier domain

Part 12 Comparison between the proposed HeldSee and the reported hyperspectral endoscopy

Part 1 Realization of the HeldSee system

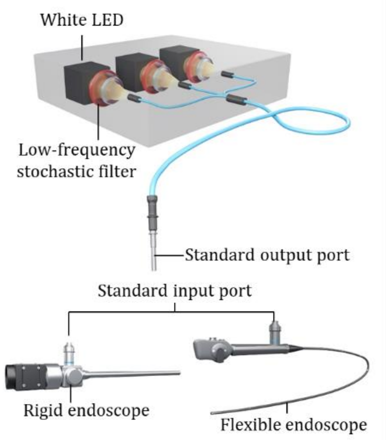

Fig. S1 Configuration of the temporal-spectrally modulated light source of HeldSee with a standard port supporting both the rigid and flexible endoscope.

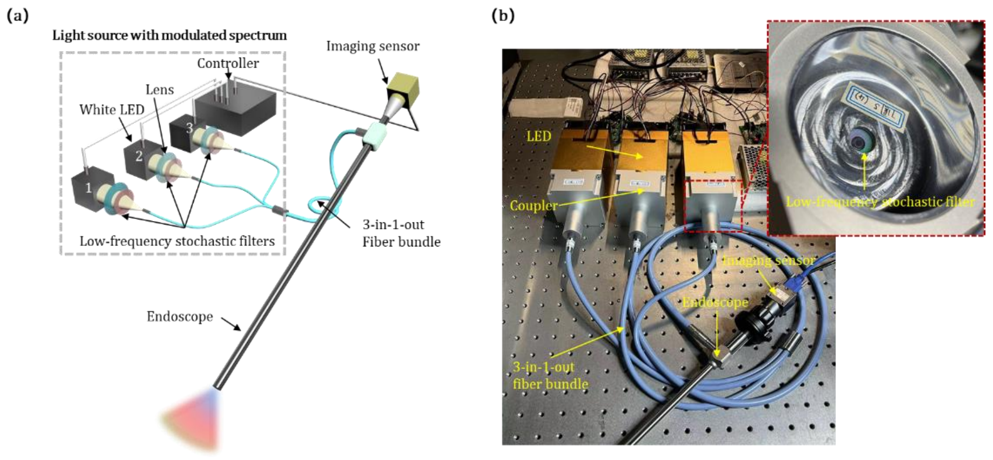

Fig. S2 Schematic **(a)** and physical **(b)** diagram of the established HeldSee prototype with a rigid endoscope as an example.

Part 2 Calibration of the prototype

2.1 Encoding spectra calibration

As demonstrated by Eq. 2 in the main text, the overall encoding function is $s(\lambda)\ f_i(\lambda)\ d_j(\lambda)$, where $i = 1, 2, 3$ and $j = 1, 2, 3$. $s(\lambda)$ denotes the illumination spectrum without the filter modulation. f_i denotes the transmission spectrum of the i_{th} filter. $d_j(\lambda)$ denotes the spectral response of the j_{th} color channel in detection.

We have used the system shown in Fig. S3 to calibrate the encoding function. Firstly, output from a monochromatic adjustable light source (Omni-λ3007i) is coupled to one end of a fiber bundle, of which the other end is connected to the endoscopy body used in HeldSee (Fig. S3a). The sample, a reflection standard (Labsphere), is illuminated by the monochromatic light, and its image is

captured by the camera. The reflected signal from the reflection standard is also collected by a spectrometer (AvaSpec-HSC-TEC). The wavelength of the monochromatic light is modulated from 400 nm by 5 nm to 800 nm, and at each step, the camera and the spectrometer make corresponding records.

The intensity of the j_{th} color channel of the image captured by the camera represents:

$$A_j = M \times W \times d_j \qquad \text{Eq. S1}$$

where M denotes the intensity of the monochromatic illumination, and W denotes the reflective spectrum of the reflection standard.

The integration of the spectrum recorded by the spectrometer represents:

$$B = M \times W \times S \qquad \text{Eq. S2}$$

where S denotes the spectral response of the spectrometer.

Secondly, the i_{th} low-frequency stochastic filtered light used in HeldSee is coupled into the endoscopy body (Fig. S3b). The reflection standard is illuminated by the low-frequency stochastic filtered light, and the reflected signal is recorded by the spectrometer, which represents:

$$C_i = s \times f_i \times W \times S \qquad \text{Eq. S3}$$

The reflective spectrum of the reflection standard, W, can be treated as a constant. A, B, and C all correct for the dark background with the light sources off. The overall encoding function $s(\lambda) f_i(\lambda) d_j(\lambda)$ can be calculated by: $sf_i d_j = A \div B \times C$.

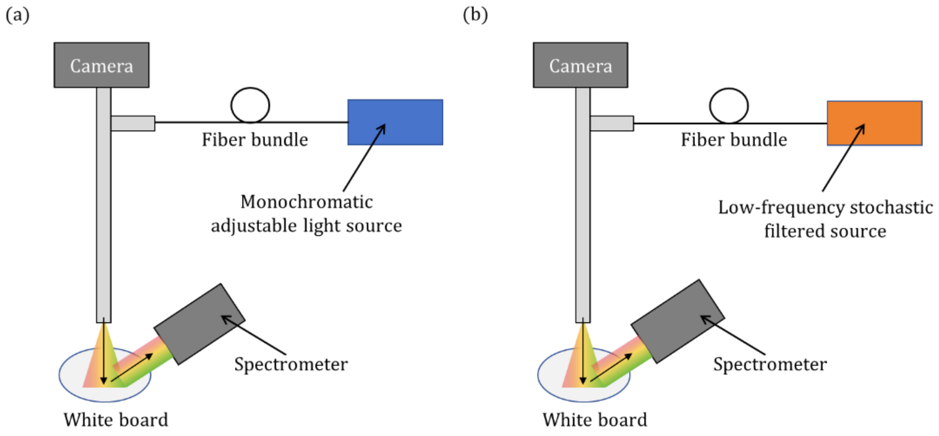

Fig. S3 HeldSee system calibration setups.

The experimentally measured C_i and A_j/B are shown in Fig. S4. Fig. S5 shows the calculated pairwise coefficient matrix of the transmission spectra of the three filters used in HeldSee system.

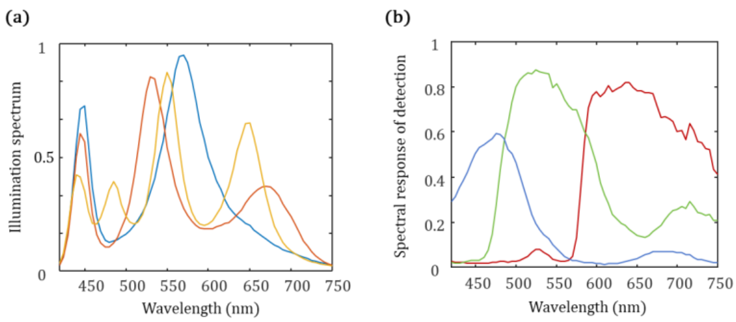

Fig. S4 Measured illumination spectrum C_i, $i = 1, 2, 3$ **(a)** and spectral response of detection A_j/B, $j = 1, 2, 3$ **(b).** The meanings of A, B, and C are presented in Eq. S1-S3.

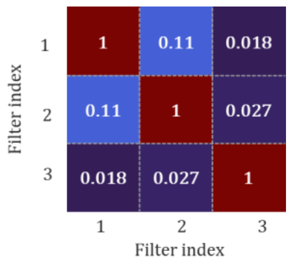

Fig. S5 Calculated pairwise coefficient matrix of the transmission function of the three filters.

2.2 Modulation speed

We have tested the prototype corresponding to a hyperspectral imaging frame rate of 66 Hz, and find that there is no crosstalk between the three raw images. In the experiment, the illumination is modulated with a period of 15 ms, and each LED is turned on for 4 ms in one period. As shown in Fig. S6, the imaging intensity when the LED1 is turned on is same regardless of whether the other two LEDs are on or off. It is stable with the modulation period number. This implies that the image captured when LED1 is turned on is immune to the interference from other modulated illuminations.

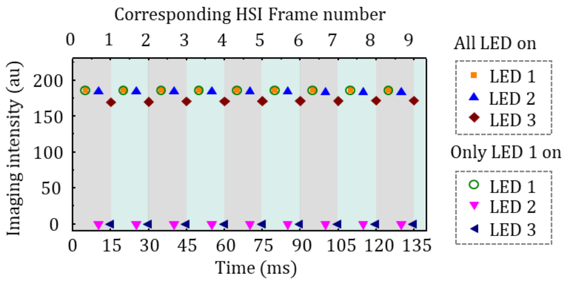

Fig. S6 Intensities of different modulated illuminations with time. The hyperspectral imaging frame number is labelled on the upper axis. “HSI” denotes “hyperspectral image”.

2.3 Spatial distribution of the modulated illumination intensity in the field of view

To test the fluctuation in the field of view for different modulated illuminations, a white board is used as the object, and the fluctuation is estimated by the spatial distribution of the three

modulated illumination intensity divided by their mean intensity, as shown in Fig. S7. The spatial distribution of the three modulated illumination are very close. The fluctuation of their relative ratio is within 5% in the whole field of view, which is considered when training the network.

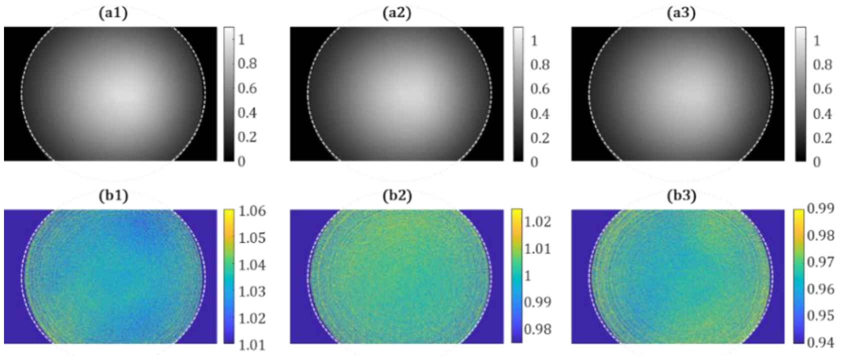

Fig. S7 (a1-a3) Experimentally measured spatial distribution of the three modulated illumination intensities in the field of view. **(b1-b3)** Spatial distribution of the three modulated illumination intensities divided by their mean intensity. The range of the color bar is limited in 0.05. The white dashed line marks the boundary of the imaging field of view of the endoscope.

Part 3 Spectral resolution of the HeldSee system

The spectral resolution of the system has been evaluated using two criteria: 1) the closest double peaks it can resolve, and 2) the bandwidth of the measured profile of a narrow peak. Thousands of spectra composed of multiple Gaussian curves with random intensities, bandwidths, and peak positions were added to the training dataset to enhance the applicability of HeldSee beyond the biological tissue spectra. It is noteworthy that, the spectral curves presented below for testing were excluded from the training dataset.

Fig. S8 shows the performance of HeldSee system to resolve double peaks with different peak-to-peak distances ranging from 15 nm to 25 nm, at different locations (500 nm, 600 nm and 700 nm) in the spectral range. HeldSee incorrectly reconstructs the double peaks of 15 nm peak-to-peak distance into one. However, it successfully resolves the double peaks of 20 nm peak-to-peak distance in its reconstructed spectra, as well as those of 25 nm peak-to-peak distance, with reasonably better contrast, demonstrating a spectral resolution of HeldSee superior to 20 nm.

Fig. S9 shows the performance of HeldSee to reconstruct the spectra for single narrow peaks of 5 nm bandwidth at different locations (500 nm, 600 nm and 700 nm) in the spectral range. To analyze the bandwidths of the reconstructed spectra, Gaussian fits are performed and the bandwidths of the fitted Gaussian profiles are all slightly less than 20 nm, in good agreement

with the resolution results tested using the double peaks shown in Fig. S8.

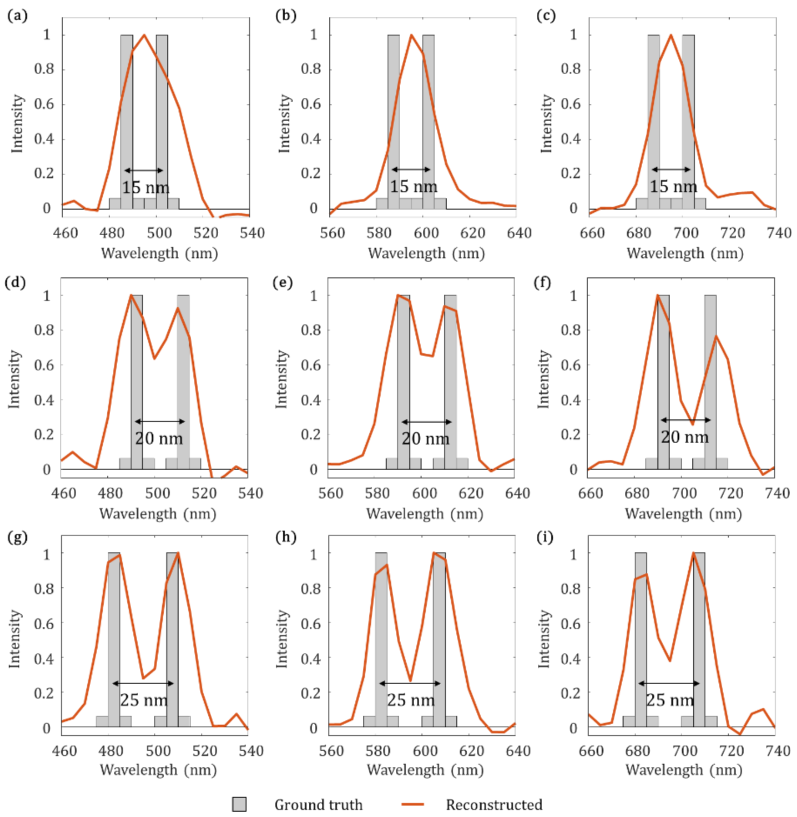

Fig. S8 The ability of HeldSee to resolve double peaks. (a-c) Ground truth (gray bars) and the reconstructed spectra of HeldSee (red lines) for the double peaks located at 500 nm, 600 nm and 700 nm, respectively, with a peak-to-peak distance of 15 nm. **(d-f)** Ground truth and the reconstructed spectra of HeldSee for the double peaks located at 500 nm, 600 nm and 700 nm, respectively, with a peak-to-peak distance of 20 nm. **(g-i)** Ground truth and the reconstructed spectra of HeldSee for the double peaks located at 500 nm, 600 nm and 700 nm, respectively, with a peak-to-peak distance of 25 nm.

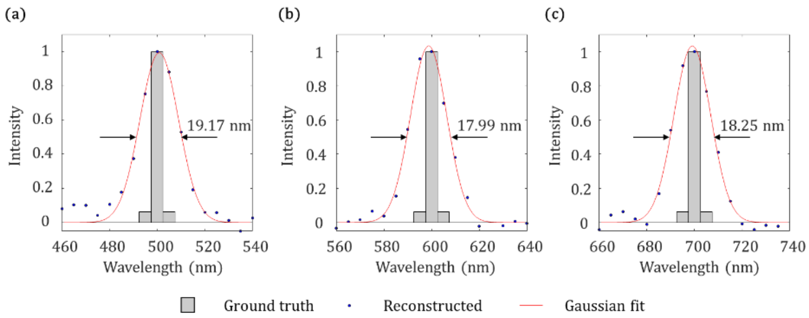

Fig. S9 The ability of HeldSee to reconstruct single narrow peaks of 5 nm bandwidth. **(a-c)** Ground truth (gray bars) and the reconstructed spectra of HeldSee (dark dots) for the single narrow peaks located at 500 nm, 600 nm and 700 nm, respectively. Gaussian fits (red lines) are performed for the reconstructed spectra to analyze the bandwidth.

Part 4 Details of collected dataset and the reconstruction network

The hyperspectral camera used to collect the spectrum dataset is a push broom camera (GaiaField-V10), with a frame rate of 0.02 fps and a spectral resolution of 2.5 nm in the range from

400 nm to 1000 nm. Although severe spatial blur exists in the captured hyperspectral images owing to the motion of living tissue, the accuracy of the spectrum can be promised because it collects the instant spectrum of a slit tissue in a snapshot manner. With a spatial scanning, the spectra in the field of view can be collected to contribute to the dataset.

The dataset contains the diffuse reflectance spectrum of various types of organs, including liver, kidney, spleen, stomach outer wall, stomach inner wall, and ear skin. The quantity of the hyperspectral image (HSI) pixels for the ear skin is 25,956,704, collected from 5 mouse ears. The spectral data for internal organs was collected from 17 mice, and the quantities for various types of internal organs are listed in Table 1. All the collected HSIs were cropped into 128×128 and 600 sub-images were randomly chosen to feed into the network for training. The batch size was set to 3, and the number of epochs was set to 300. Optimizer used was ADAM, with β_1 set to 0.9 and β_2 set to 0.999. Mean square error (MSE) was used as the cost function. An initial learning rate of 4×10^{-4} was used, and cosine annealing was applied to gradually reduce it to 1×10^{-4} by epoch 300.

Table S1 Quantity of the spectrum data for internal organs

Organ type	Quantity of pixels
Spleen	1,764,633
Stomach outer wall	2,565,282
Stomach inner wall	3,181,553
Liver	15,222,183
Kidney	9,919,177

Integration of the encoding spectra in the attention module can increase the reconstruction accuracy. Fig. S10 shows the spectrally averaged relative absolute error (RAE) achieved using the same framework in Fig. 2 but different attention module with or without the encoding spectra input for guidance. Lower RAE has been enabled by the proposed ESANet with the encoding guided spectral attention module.

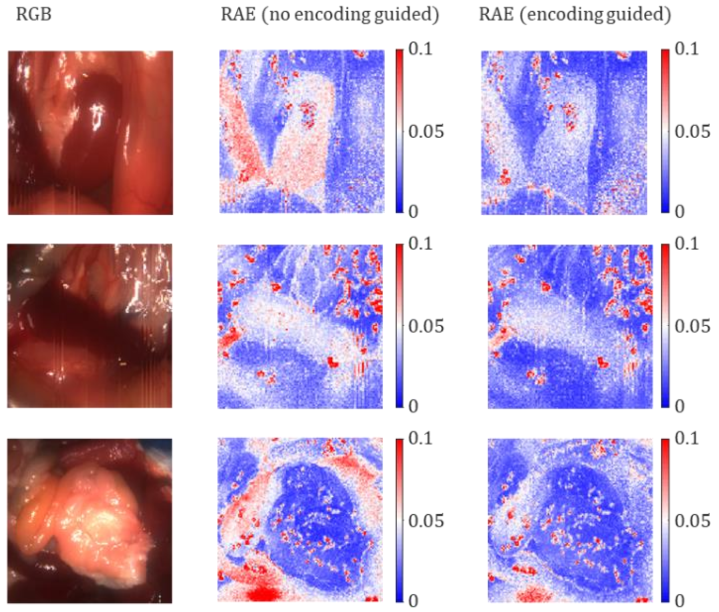

Fig. S10 The RAE achieved by the networks with and without encoding guidance in the attentional module. The RGB view sights are synthesized from the GT HSI cube. The measurements for reconstruction contain 5% detection noise and 5% intensity variation of between different modulated illuminations.

Motion artefacts exist in the *in vivo* hyperspectral image dataset collected using the push broom camera. However, in the implementation of HeldSee, the capture time per hyperspectral imaging frame is less than 50 ms and the actual encoded images used to reconstruct the hyperspectral images are free of motion artefacts. Fortunately, the reconstruction error in motion-artifact-free cases is low, and no visible artefacts appear in the reconstructed hyperspectral image (as shown by the results in Fig. 3 in the main text), owing to the adaptability of the neural network and the usage of small convolutional kernels and shallow spatial convolutional layers. Even when severe motion artefacts exist somewhere, the impact range of the artefacts is very limited. As shown in Fig. S11a is a synthesized color image of an *in vivo* liver from our HSI dataset, with severe artefacts caused by the *in vivo* motion and high specular reflection (in the blue square). It can be observed that the high error occurs very close to the line-shaped artefacts and regions of high specular reflection. The RAE remains low (< 5%) in areas without artefacts (in the yellow square in Fig. S11). Therefore, motion artefacts do not pose a significant challenge in this study.

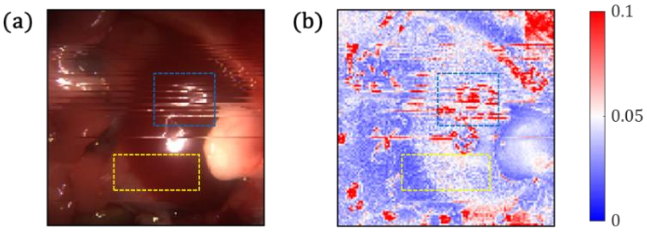

Fig. S11 Impact of the artefacts on the reconstruction accuracy. **(a-b)** Synthesized color image of an *in vivo* liver from our dataset (a), and the corresponding reconstruction RAE distribution (b). Blue square highlights regions with artefacts. Yellow square highlights regions free from artefacts and with low reconstruction error.

Part 5 Signal-to-noise ratio comparison between HeldSee and conventional scanning approach

We have performed an experiment to compare the signal-to-noise-ratio (SNR) between the proposed HeldSee and conventional wavelength-scanning approach. In the two approaches, a same endoscope equipped with a color camera was used for imaging, while the illumination modulation way was different. In the wavelength-scanning approach, the radiation from a xenon lamp is modulated by a monochromator (model number: Omni-λ3007i) to enable narrow band (NB) illumination modulation. The central wavelength of the NB illumination is modulated from 420 nm to 750 nm, with a step size of 20 nm. The illumination intensity of the NB illumination corresponding to different central wavelength, as well as the intensity of the 3 modulated illuminations used in HeldSee are shown in Fig. S12a. Fig. S12b shows ratio between the 3 modulated illuminations used in HeldSee and the peak intensity of the NB illuminations, with most values less than one, demonstrating that the peak intensity of the NB illuminations were mostly larger than the intensity of the modulated illuminations used in HeldSee at the corresponding central wavelengths.

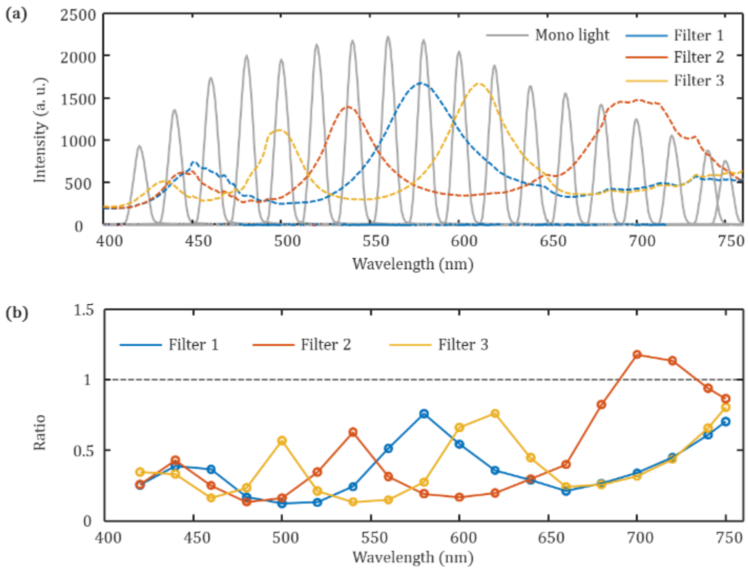

Fig. S12 (a) Spectra of the illuminations used in HeldSee (blue, red and yellow lines) and the conventional wavelength-scanning approach (Gray lines). 18 illuminations with different central wavelength were used. **(b)** Ratio between the 3 modulated illuminations used in HeldSee and the peak intensity of the NB illuminations.

In the experiment, the sample is a motionless stomach outer wall of a mouse just died so the hyperspectral image achieved by the conventional wavelength-scanning. approach with low frame rate is free from motion blur. Figs. S13a-b show the hyperspectral image in the format of synthesized RGB, achieved using HeldSee and the wavelength-scanning. approach, respectively. The number of noise points achieved by HeldSee are less than that achieved using the wavelength-scanning. approach, demonstrating the better SNR of HeldSee than the conventional method. Fig. S13c shows the spectra achieved by HeldSee and the conventional wavelength-scanning approach, respectively, which appear to be similar. While the spectra achieved by wavelength-scanning approach are more vulnerable to noise, especially at the short wavelengths. The 2D images at various spectral channel achieved using HeldSee also present better SNR, as shown in Fig. S14.

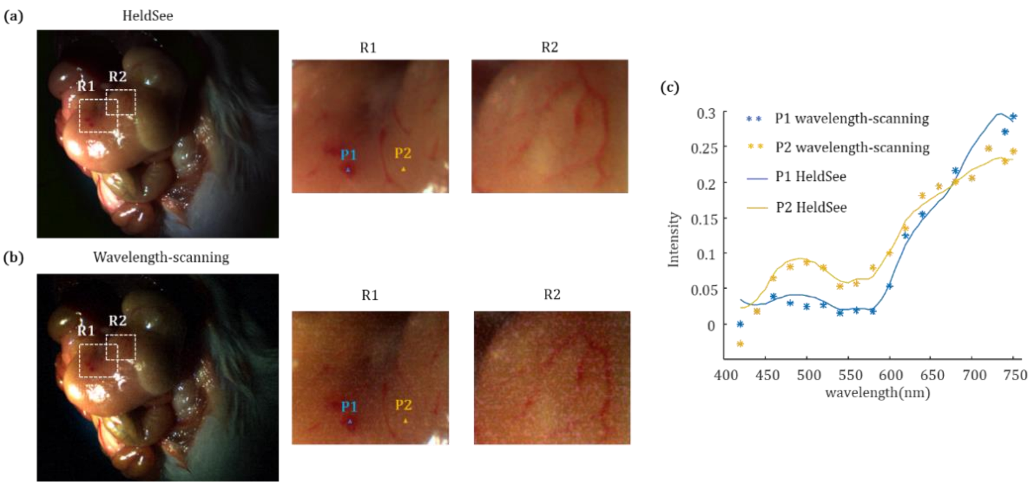

Fig. S13 (a-b) RGB image synthesized from the hyperspectral image achieved by HeldSee (a) and the conventional wavelength-scanning. approach (b). Two regions (R1 and R2) are enlarged for a clear show. **(c)** The spectra at two points labeled in (a-b) achieved by HeldSee and the conventional wavelength-scanning. approach.

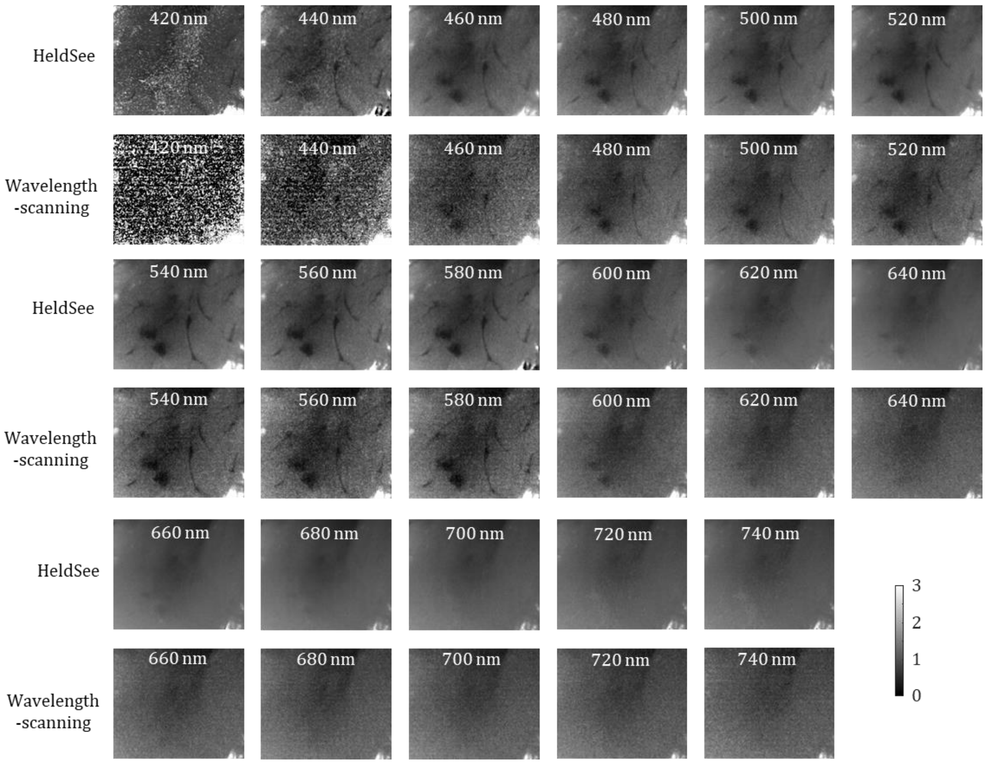

Fig. S14 Images at various spectral channel, achieved by HeldSee and the conventional wavelength-scanning. approach respectively.

Part 6 Reconstruction accuracy with low-frequency and conventional stochastic filters

The spectrum reconstruction precision relates with the transmission spectra of the filters used to modulate the illumination. We have trained corresponding networks and compared the spectrum reconstruction precision with two kinds of temporal spectrally modulation functions, which are the low-frequency stochastic modulation (Fig. S15a) and conventional stochastic modulation with

random spectra (Fig. S15b), both with the modulation number of 3. The achieved reconstruction precisions are shown in Fig. S16. The result demonstrates that the low-frequency stochastic modulation can achieve the smaller mean squared error (MSE) in the reconstruction at diverse noise level, thanks to its larger Fourier amplitude in the frequency range where the diffuse reflectance spectra of tissue sample dominate (Fig. 1e in the main text).

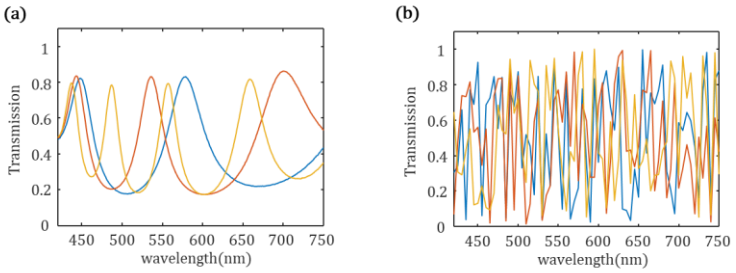

Fig. S15 Low-frequency stochastic modulation function **(a)** and conventional stochastic modulation function **(b)**.

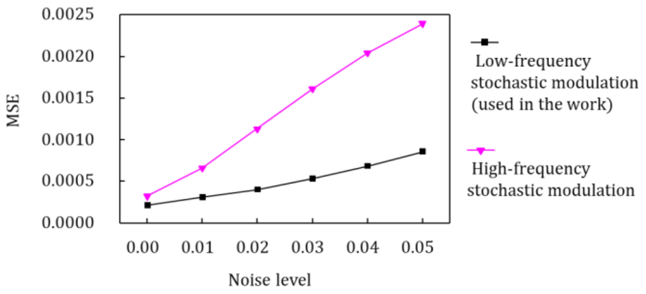

Fig. S16 Reconstruction MSE with the low-frequency and high-frequency stochastic modulation.

Part 7 Reconstruction accuracy with increased modulation number

It is possible to increase the reconstruction accuracy by using more encoding numbers. We have investigated the MSE of reconstruction when the 4th and the 5th filter were introduced. The transmittance functions of the new filters are shown in Fig. S17.

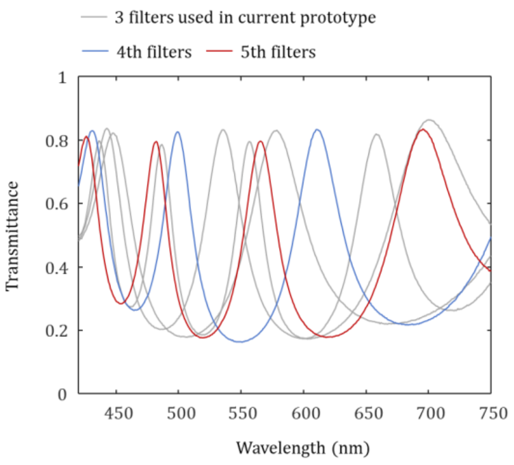

Fig. S17 Transmittance curves of the introduced 4th and 5th low-frequency stochastic filters. The transmittance curves of the 3 filters used in our original prototype are shown by the gray lines.

Given the same consistency in illumination intensity distribution and noise level of individual encoded images, an increase of the modulation number leads to a lower MSE and thus higher reconstruction accuracy, as shown in Fig. S18. In practice, if the overall frame rate of hyperspectral imaging is to be maintained—that is, keeping the total exposure time for all encoded images constant—increasing the number of modulations can degrade the SNR of individual encoded images due to the shorter exposure time per modulation. Besides, maintaining intensity consistency for all the modulated illuminations becomes increasingly challenging with a higher modulation number. Thus, the overall effectiveness of using more encoding requires a careful evaluation.

When the intensity ratios between differently modulated illuminations fluctuate more severely in the field of view, the MSE increases (Fig. S18a). When the fluctuation ratio exceeds 5%, the MSE increases slowly and gradually stabilizes at a steady value. In such case, a higher modulation number leads to a lower MSE, even at higher fluctuation rates. Consequently, the fluctuation ratio or the consistency level, becomes a minor factor to consider when try to introduce more modulations.

By comparison, noise is a more significant factor. The positive impact of using more modulations on reconstruction accuracy can be negated by the negative effect of increased noise level. For example, assuming the exposure time of the individual encoded image is 10 ms when the modulation number is 3, then the exposure time should be suppressed to 6 ms to maintain the final frame rate when the modulation number is increased to 5, which would increase the noise level by 1.6 times approximately. Here, we simply assume that the noise ratio is inversely proportional to the exposure time. The MSE for the modulation number of 5 is not necessarily lower than the MSE achieved by the modulation of 3, considering the noise level increases by 1.6 time (Fig. S18b). To effectively improve the reconstruction accuracy with a higher modulation number, the light source power can be increased to maintain the SNR of each encoded image with shorter exposure time. Consequently, it is important to comprehensively evaluate practical factors such as the available light source energy, expected frame rate, and cost when considering further upgrades.

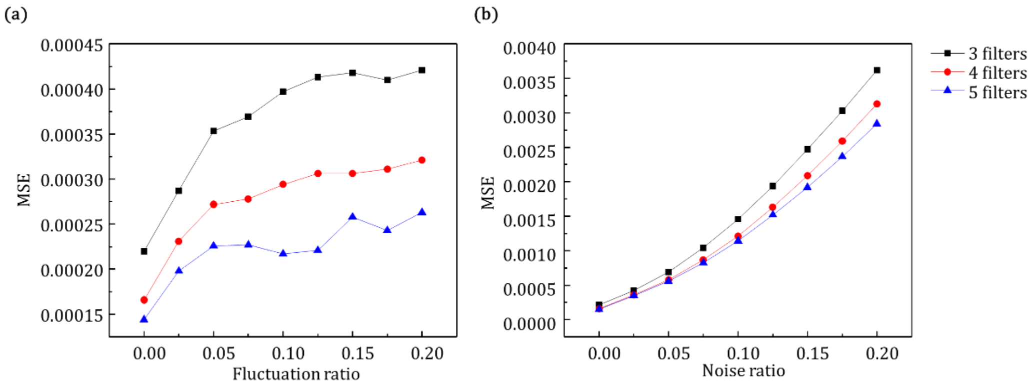

Fig. S18 Reconstruction MSE corresponding to the modulation number of 3, 4, and 5 respectively, at different fluctuation ratio between modulated illuminations in the field of view (a) and at different noise level of individual encoded images (b).

Part 8 Narrow band imaging function of HeldSee

Narrow band endoscopy (NBE) shows tissue images of a narrow spectral band of 415 nm or 540 nm, where hemoglobin exhibits strong absorption, effectively enhancing the visibility of blood-vessel-like structures. The image at 540 nm can be extracted from the HSI achieved by HeldSee system, without the need for an additional NB light source. As shown in Fig. S19, the image at 540 nm (NBE) effectively enhances the contrast of liver features, compared to the full-spectrum visible-band image (white light endoscopy).

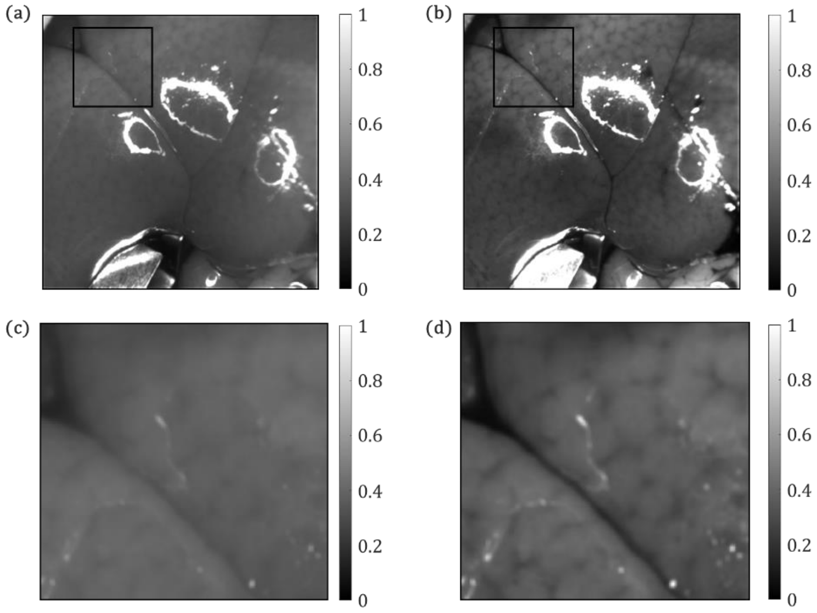

Fig. S19 Images of a clamped liver in ischemia, generated from the HSI. **(a)** Sum of all the spectral channels within the visible band (420-700 nm). **(b)** Image at spectral channel of 540 nm. **(c-d)** Magnified views of regions in the black squares in (a-b).

Part 9. Monte Carlo calculation model

Monte Carlo (MC) calculation was performed to generate a lookup table for blood oxygen saturation (sO_2) and blood volume fraction (B) based on the reconstructed spectral data. The liver tissue is assumed to be a uniform semi-infinite block. The ear is an infinite block with a thickness of 5mm. In calculation, the photons penetrating the ear don't return considering the ear is pressed tightly against a piece of black cloth in our experiment. The refractive index of biological tissue is set to be 1.4. The incident angle is set to be within π/10. Photons with wavelength from 450 nm to 700 nm are incident into the tissue, being absorbed and scattered during the propagation, and those ultimately scattered out of the tissue towards the detector, with their remaining energy above $1e^{-4}$, in a surficial area with a radius of 6 mm, are recorded to calculate the diffuse reflectivity.

The diffuse reflective spectrum can be determined by the MC calculation given the scattering and absorption coefficient, which is related with sO_2 and B, so a lookup table relating the spectral data and the tissue compositional information (sO_2 and B) can be established. The tissue's scattering coefficient (unit: cm^{-1}) is: $\mu_s = a(\lambda / 500nm)^{-b} / (1\text{-}g)$, and the parameters of *a*, *b*, and *g* for various organs are set according to the reference (*1*). For liver, *a*=9.2; *b*=1.05 and *g* =0.95. For ear skin, *a*=30.6; *b*=1.1 and *g* =0.95. The tissue's absorption coefficient (unit: cm^{-1}) is: $\mu_a = \sum_{j=1,2} \ln(10) \cdot f_{v,j} \cdot \mu_{a,j}$, in which $f_{v,j}$ and $\mu_{a,j}$ denote the number of moles and molar extinction coefficient of oxyhemoglobin (*j*=1) and deoxyhemoglobin (*j*=2) respectively. $\mu_{a,j}$ is obtained from reference (2) (unit: $cm^{-1}M^{-1}L$) and $f_{v,1} = B \cdot sO_2 \cdot C / (64500g \cdot M^{-1})$, $f_{v,2} = B \cdot (1 - sO_2) \cdot C / (64500g \cdot M^{-1})$, in which C is the mass concentration within blood (set to be a typical value of 150g/L). In the lookup table, the sO_2 is scanned from 0.3 to 0.9 and B is scanned from 0.01 to 0.1 for the liver tissue. For the skin the sO_2 is scanned from 0.05 to 0.95 and B is scanned from 0.0025 to 0.04.

Part 10 Dependence of the composition calculation accuracy on the spectral channel number

The accuracy of composition calculation could be improved by using spectrum with more spectral channel, which is enabled by a broader spectral range or a smaller step interval. We have investigated the composition calculation accuracy in simulation for oxyhemoglobin, deoxyhemoglobin, fat and water, based on four kinds of spectral parameters: 1) range: 420 nm to

750 nm, step: 5 nm (the condition of our current prototype); 2) range: 420 nm to 1000 nm, step: 5 nm (spectral coverage broader than the current condition), 3) range: 420 nm to 750 nm, step: 2.5 nm (step interval smaller than the current condition), and 4) range: 420 nm to 1000 nm, step: 2.5 nm (spectral coverage broader and step interval smaller than the current condition).

The relative blood volume, oxygen saturation, and relative volume of water or fat of a liver-like tissue (of which the reduced scattering coefficient is set to $9.2\left(\frac{\lambda}{500nm}\right)^{-1.05}$, based on data in reference (*1*)) are calculated by comparing a noisy spectrum with the data in a lookup table. The added noise is Poisson noise, with its level inversely relates to the photon count. The lookup table relating composition to diffuse reflection spectra are established based on the Beer-Lambert Law. The larger the noise, the more likely an incorrect value will be found in the lookup table. The composition calculation errors are averaged for a serious of tissue with the relative blood volume = [0.02 by 0.02 to 0.5], oxygen saturation = [0.05 by 0.05 to 1], relative volume of water = [0.05 by 0.05 to 0.7] and relative volume of fat plus water = 0.7. The averaged errors at various photon counts are plotted in Fig. S20 for the four types of spectral parameters. The results can be summarized as follows.

1) Given the same spectral coverage, reducing the spectral step interval from 5 nm to 2.5 nm does not improve compositional calculation accuracy, and may even degrade it when the photon budget is quite low due to the reduced photon count per channel at smaller step intervals.

2) With the same spectral step interval, extending the spectral range from 420–750 nm to 420–1000 nm can improve compositional calculation accuracy, particularly for water and fat, which exhibit stronger absorption in the infrared region.

Consequently, to improve the quantitative detection accuracy in clinical condition, it would be more effective to extend the spectral range rather than increase spectral resolution in the future.

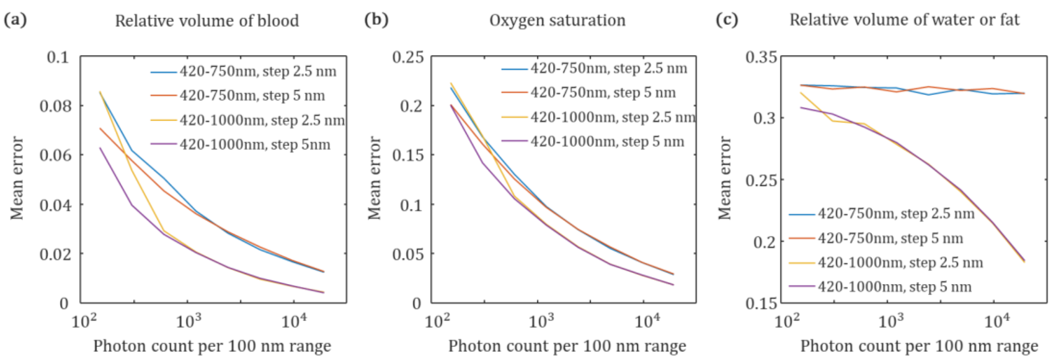

Fig. S20 Calculation error in the relative blood volume (a), oxygen saturation (b), and relative volume of water or fat (c) corresponding to different number of photons incident on the tissue, based on the diffuse reflectance spectra with varying spectral ranges and step intervals. The vertical axis represents the averaged composition calculation errors for a serious of tissue with the relative blood volume = [0.02 by 0.02 to 0.5], oxygen saturation = [0.05 by 0.05 to 1], relative volume of water = [0.05 by 0.05 to 0.7] and relative volume of fat plus water = 0.7. As the total amount of water and fat remains constant in our simulation, they exhibit the same compositional calculation error.

Part 11. Encoding analysis in the Fourier domain

The intensity of the encoded image is

$$I = \int e(\lambda) \cdot t(\lambda) d\lambda \qquad \text{Eq. S4}$$

where e denotes the encoding spectrum, including the illumination and detection spectral modulation. t denotes the spectrum of target sample.

Substitute $e(\lambda) = \int E(v_1)\exp(i\lambda v_1)dv_1$ and $t(\lambda) = \int T(v_2)\exp(i\lambda v_2)dv_2$ to Eq. S4, where E and T denote the Fourier transform of the encoding spectrum and target spectrum, respectively. Then we get,

$$\begin{aligned} I &= \int_{v_1}\int_{v_2}\int_{\lambda} E(v_1)\cdot T(v_2)\cdot \exp[i(v_1+v_2)\lambda]dv_1 dv_2 d\lambda \\ &= \int_{v_1}\int_{v_2} E(v_1)\cdot T(v_2)\{\int_{\lambda}\exp[i(v_1+v_2)\lambda]d\lambda\}dv_1 dv_2 \end{aligned} \qquad \text{Eq. S5}$$

The integral inside the curly brackets is non-zero only when v_1=-v_2, then

$$\begin{aligned} I &= A\int_{v_1}\int_{v_2} E(v_1)\cdot T(v_2)dv_1 dv_2\Big|_{v_1=-v_2} \\ &= A\int_{v} E(-v)\cdot T(v)dv \end{aligned} \qquad \text{Eq. S6}$$

where A denotes a constant.

As the encoding spectrum is real, $E(-v) = E^*(v)$, then we get,

$$I = A\int_{v} E^*(v)\cdot T(v)dv \qquad \text{Eq. S7}$$

Consequently, the detection intensity is the weighted sum of the Fourier components of the spectrum of target sample, with the weight equal to the conjugate of the Fourier transform of the encoding function.

Part 12. Comparison between the proposed HeldSee and the reported hyperspectral endoscopy

Table S2 Comparison between the proposed HeldSee and the reported hyperspectral endoscopy

**Work**	**Mechanism**	**Time cost to capture one frame of HSI***	**Resolution of HSI***	**Spectral channel**	**Testing scene**
J. Biophotonics 10, 2017, 553-564.	Narrow band scanning by switching mono LED	0.45s for 2D imaging	350*370	6	In vivo tissue (no obvious texture feature shown).
Med. Image Anal. 48, 2018, 162-176.	Output of a fiber bundle being spatially reshaped to 1D and then dispersed for one snapshot detection)	~83ms for 2D imaging	171	24	In vivo tissue (no obvious texture feature shown).
Nature Commun. 10, 2019, 1902	Single line HSI diffused with RGB image in a push broom process	~50ms for a single line	10000	Several hundreds	Phantom & Ex vivo tissue with no obvious texture feature.
J Biophotonics 14, 2021, e202100078	Single line HSI diffused with RGB image in a push broom process	~92ms for a single line	10000	160	In vivo tissue (while the texture feature shown is severely blurred).
Opt. Express 32, 2024, 16090	Spectrally scanning based on AOTF	390s for 2D imaging	-	186	Phantom
Sci. Adv. 9, 2023, eadd6778	Snapshot multispectral camera	40ms for 2D imaging	272 × 512	16	In vivo tissue with clear features
**HeldSee**	**Spatial-temporal spectral encoding approach based on low-frequency stochastic filters**	**~50ms for 2D imaging (15ms is possible)**	**1920×1200 Full HD**	**67**	**In vivo tissue with capillaries (diameter ~37 um) clearly shown.**

* “HSI” denotes “hyperspectral image”.

Reference

1. S. L. Jacques, Corrigendum: Optical properties of biological tissues: a review. *Phys. Med. Biol.* **58**, 5007-5008 (2013).
2. https://omlc.org/spectra/hemoglobin/summary.html.
